# Surgical Cases

**Published:** 1849-01

**Authors:** J. M. Steiner

**Affiliations:** Assistant Surgeon U. S. Army


### MEDICAL EXAMINER,

AND

### RECORD OF MEDICAL SCIENCE.

NEW SERIES.—NO. XLIX.—JANUARY, 1849.

### ORIGINAL COMMUNICATIONS.

*Surgical Cases.* By J. M. Steiner, M. D., Assistant Surgeon U. S. Army. (Communicated in a letter to one of the Editors.)

Three or four days after the battle of Cerro Gordo, I volunteered my services to the Surgeon General to return to Plan del Rio, whither the most of our wounded had been conveyed after the action. Among others placed under my charge by the Senior Medical Officer, Dr. Cuyler, was an Illinois volunteer by the name of Todd, with the worst looking wound I have ever beheld.

This man was struck by a cannon ball, which carried away the left half of the inferior maxillary bone, stripping the soft parts from the superior maxillary as high up as the malar bone, tearing away the soft parts from the left side of the neck to within an inch and a half of the clavicle, laying bare the transverse processes of the second and third cervical vertebræ, and exposing the external carotid with the most of its branches. The wound, when I first saw it, was in a miserable condition, covered with fragments of the lacerated tissues, already gangrenous, and pouring forth an ill-conditioned sanies, the smell of which was intolerable. The constitutional symptoms corresponded with the condition of the wound. The pulse was small and corded, and exceedingly frequent, the tongue furred and dry, the skin hot, and harsh to the touch, and the bowels torpid. I dissected away the partially loosened sloughs which covered the wound, and applied an emollient poultice. For the relief of the constitutional disturbance a draught of salts was given, which having acted briskly upon the bowels, abated the fever, and the patient expressed himself much relieved. On the following day the appearance of the wound was much improved, the pus assumed a more healthy character, and the man's spirits began to revive. The third day after my arrival he was conducted on a litter to the general hospital at Jalapa, where I continued in charge of him, at the special request of Dr. Hill, then in charge of the volunteer division of the hospital. The treatment at Jalapa consisted exclusively in the application of the olive oil dressing, with proper attention to the regulation of the bowels, and the mild exhibition of opiates at night. Under this course he improved daily, the wound commenced granulating, and by the first of June he had nearly recovered, a small fistulous orifice communicating with the mouth alone remaining unclosed. As a different arrangement was made at this time by the Senior Surgeon in the assignment of the wards, Todd fell into the hands of another surgeon, and I saw no more of him for some time. At the end of two weeks, I think on the 15th of June, I was placed in charge of the sick and wounded, who were, by the order of the General-in-Chief, to remain at Jalapa, as they were deemed too unwell to be removed to Perote, where all, whose lives would not be manifestly endangered by the transportation, were being carried. To my surprise I found Todd among the number left under my charge. Upon an examination of his wound, I found it in a worse condition than when I first saw him at Plan del Rio. The neck was enormously swollen, the whole of the newly formed granulations in a sloughing condition, and a dirty, ill-conditioned pus running abundantly from the wound. The constitutional symptoms were similar to those at Plan del Rio. I applied to the wound a large emollient poultice, and administered 10 grs. of blue mass, followed in a few hours by a draught of the sulphate of magnesia. The next morning I thought it desirable to repeat the poultice, with the view of cleansing the wound and lessening the inflammation.

On the afternoon of the same day,—the 17th—I was hastily informed by the steward, while at my quarters, about 100 yards distant from the hospital, that Todd was bleeding to death. Upon arriving in the ward a few moments after, I found the poor fellow bathed in blood. Stripping the poultice from his neck, I tore away with my hand the gangrenous flesh which concealed the artery, when I was blinded by the gush of blood from the external carotid, which was the vessel ruptured. Pressing the right hand with all my force below the part ruptured, I succeeded in arresting the hemorrhage. The poor fellow was so much exhausted that I was obliged to turn him almost upon his head, in order that his brain might have the benefit of what little blood yet remained in his body.

Having with care substituted the strong hand of an attendant in lieu of my own, in controlling the hemorrhage, I made an incision from the left sterno-clavicular articulation, extending obliquely upwards and backwards two inches in length, with a view of ligating the carotid artery. The neck was so enormously enlarged, and the tissues so much altered, that I encountered more difficulty in effecting my object than I expected. I encountered the vessel, however, and having carefully separated it from the vein and nerve, succeeded without trouble in ligating it. Upon measurement, I found the depth of the incision an inch and three-quarters. For two days he was kept regularly on wine, and boiled milk and toast. Two days after, all the sick were removed to Perote. The few days I was at this place Todd rapidly improved. I left Perote ten days after the performance of the operation, for the city of Puebla, but have learned since, through several sources, that the man is entirely well, and only waiting an opportunity to leave for home.

The other case fell under my notice at the battle of Chapultepec. The man's name was Hall, and belonged to Company K, 14th Regiment of Infantry. He was struck by a round shot just below the trochanter major of the left thigh, which tore the bone and soft parts into fragments for some inches below. The upper portion of the femur was longitudinally fractured as far as the joint. Having consulted with Dr. Hagan, of the 14th Infantry, with regard to what should be done, we both concluded that the only chance of life, which the poor fellow had, depended upon his thigh being taken off at the hip joint. Having stimulated him sufficiently by the administration of brandy to stand the shock of the operation; I had him placed upon a long low table, which, fortunately for my purpose, I found in the building Molino del Rey, near at hand. I made use of four assistants. Two were so placed as to restrain the motions of the arms, one seated upon the ground, with his back to the table, held quiet the right lower extremity; while another stood to my right, for the purpose of compressing the flap as it was cut. Dr. Hagan took charge of the injured extremity, and was of much use to me in the operation. The man had a very large thigh, and I was obliged to operate with a knife but six inches in the blade. I overcame this difficulty, however, as you will presently see, by a very simple manœuvre. Commencing about midway between the anterior superior spinous process of the ilium and the trochanter major, I thrust the knife inwards and slightly downwards across the anterior surface of the neck of the femur the full length of the blade, when, by depressing the handle, at the same time slightly withdrawing the blade, I enlarged the point of entrance to the extent of two inches, which admitted with facility a portion of the handle of the catlin, enabling me, by making slight pressure upon the inner portion of the thigh, to transfix it, when, by depressing the extremity of the blade, and elevating the handle, I enlarged the point of exit until it equalled in size that of entrance. My attendant then inserted the two front fingers of both hands into each orifice of the incision, overlapping the thumbs above, and, by compression, so narrowed the width of the flap that I was enabled to cut it with my short knife without difficulty, while the same manœuvre answered in controlling the hemorrhage. The anterior flap having been retracted by the assistant, I drew the edge of the catlin across the anterior surface of the capsular ligament, when, by grasping with my left hand the extremity of the fractured bone, and forcibly pressing it downwards, I succeeded in rupturing the ligamentum teres, and the head of the bone started from the socket. Placing the blade of the catlin between the socket and the inferior surface of the neck of the bone, which, with the aid of my left hand, I gently rotated inwards, so as to avoid the trochanter major, I cut the lower flap. The operation did not occupy me more than twenty-five seconds. The loss of blood was inconsiderable, yet the prostration that ensued was excessive. The arteries were all ligated, the wound dressed, and reaction brought about by the administration of brandy and ammonia. I should like to have stayed by this man and nursed him myself; for I really believe, could I have done so, he would now be alive; but my duties called me elsewhere, numbers of wounded were suffering for the want of medical assistance, and I was obliged to leave Hall in other hands. He survived only until evening.

